# Rubella and congenital rubella syndrome in the GCC countries: A retrospective ecological analysis of elimination progress and scenario-based projections, 2020–2024

**DOI:** 10.1016/j.jve.2026.100636

**Published:** 2026-09-23

**Authors:** Salah T. Al Awaidy, Amani Abdelmoniem Mahmoud Mustafa, Sumaiya Al Aamri, Jameela alsalman, Hamad Eid H.A. Al-Romaihi, Khalid Hamid Elawad, Mahim Albalushi, Rana Al Maghrabi, Ahmed AlHammadi, Parvaiz A. Koul, Ozayr Mahomed

**Affiliations:** a Freelance Public Health Senior Consultant, Muscat, Oman; b Health Protection and Communicable Disease Control Department, Ministry of Public Health, Doha, Qatar; c Disease Surveillance Department Center of Diseases Control and Prevention, MOH, Muscat, Oman; d King Hamad American Mission Hospital, Manama, Bahrain; e Health Protection Management, PHC Cooperation, Doha, Qatar; f Injury Management, Gulf CDC, Riyadh, Saudi Arabia; g Pediatric Infectious Diseases Division, Sultan Military Medical City, Al Faisal University, Riyadh, Saudi Arabia; h Department of Infectious Diseases, Integrated Hospital Care Institute, Cleveland Clinic Abu Dhabi, Abu Dhabi, United Arab Emirates; i Department of Internal and Pulmonary Medicine Department, Sheri Kashmir Institute of Medical Sciences, Srinagar, India; j Discipline of Public Health Medicine, University of KwaZulu Natal, South Africa

**Keywords:** Rubella, Congenital rubella syndrome, CRS, Epidemiology, Migrant health, Immunization, Elimination, Incidence, Gulf Cooperation Council countries, GCC countries

## Abstract

**Introduction:**

Rubella and congenital rubella syndrome (CRS) remain vaccine-preventable priorities. Although the Gulf Cooperation Council (GCC) countries maintain high routine rubella-containing vaccine (RCV) coverage and well-established immunisation programmes, the region is also characterised by large migrant populations, intense international mobility, and complex population immunity profiles that may sustain vulnerability to rubella transmission even in high-coverage settings. This study assessed progress toward rubella and CRS elimination in the GCC during 2020–2024 and examined the policy implications of the observed patterns.

**Methods:**

A retrospective ecological analysis conducted using aggregated country-level rubella surveillance, CRS, and immunization data from the six GCC countries for 2020–2024. Rubella incidence per 1,000,000 population was calculated and compared with the WHO elimination threshold of less than 1 case/1,000,000/year. Scenario-based Poisson regression models projected annual reported cases for 2026–2055 under four vaccination coverage scenarios: baseline, optimistic, pessimistic, and disruption.

**Results:**

Bahrain and Oman maintained zero rubella incidence with sustained two-dose coverage of 97–99%. Kuwait reported six total cases (peak incidence: 0.7/1,000,000). Qatar recorded 16 cases in 2023 (incidence: 5.4/1,000,000) despite three consecutive years of 99% two-dose coverage. Saudi Arabia reported 476 total cases (peak incidence: 5.5/1,000,000) despite 96–98% RCV1 coverage. The UAE reported 67 total cases with a persistent non-zero burden. No CRS cases were reported region-wide. Pooled GCC countries rubella incidence did not meet the WHO elimination threshold in any study year, peaking at 4.7/1,000,000 in 2023. Under the optimistic scenario, all countries are projected to reach zero cases by 2040.

**Conclusions:**

The GCC countries have achieved substantial rubella control, with two member states demonstrating elimination-consistent epidemiological patterns. However, high childhood coverage alone did not prevent episodic transmission in Qatar, Saudi Arabia, and the UAE, reflecting residual immunity gaps. Sustaining regional elimination requires targeted strategies for adult migrant populations, strengthened active CRS surveillance, and a coordinated regional importation response.

## 1 Introduction

Rubella is a mild febrile exanthem in immunocompetent children and adults,^1^ but its public health significance is defined by infection occurs during pregnancy.^2^ First-trimester maternal infection carries a risk exceeding 85% of fetal damage, resulting in miscarriage, stillbirth, or congenital rubella syndrome (CRS).^3^ CRS is characterised by a constellation of severe and irreversible birth defects classically comprising sensorineural deafness, congenital cataracts, and structural cardiac malformations.^4^^,^^5^ Because a substantial proportion of rubella infections are subclinical or clinically nonspecific, many mothers of CRS-affected infants do not recall any recognisable illness during pregnancy.^5^ Furthermore, CRS remains under-ascertained in settings where confirmatory laboratory capacity is limited.^2^These clinical and epidemiological features make rubella control an enduring priority in maternal, child, and infectious disease public health systems.

Global progress in rubella control over the past decade has been substantial. Reported rubella cases declined from 93,816 in 2012 to 17,407 in 2022,^2^ accompanied by expanded introduction of rubella containing-vaccines (RCV) into national immunisation schedules, which increased from 132 to 175 countries over the same period.^6^ Rubella and CRS elimination targets remain embedded within the World Health Organization (WHO) Immunisation Agenda 2030.^6^ and the Region of the Americas achieved verified elimination in 2015, demonstrating that this goal is operationally attainable.^7^ Nevertheless, progress is geographically uneven. Low-incidence settings remain vulnerable to importation-driven resurgence when immunity gaps persist, and programmatic disruptions, including those associated with the COVID-19 pandemic, have eroded coverage gains in several regions.^2^^,^^7^

The six Gulf Cooperation Council (GCC) countries Bahrain, Kuwait, Oman, Qatar, Saudi Arabia, and the United Arab Emirates represent a regionally important and epidemiologically distinctive case study. A single dose of a rubella-containing vaccine confers an estimated 97% vaccine efficacy and provides lifelong immunity. Across the GCC, rubella-containing vaccines (as MR or MMR) were incorporated into national schedules between the late 1980s and early 1990s, supplemented in several member states by historical mass catch-up campaigns to close population immunity gaps across multiple birth cohorts. All the six states have incorporated the measles-mumps-rubella (MMR) vaccine into national childhood immunization schedules, and WHO/UNICEF estimates of national immunization coverage indicate sustained high two-doses RCV coverage (>95%) among children under two years of age across most member states over the last decade.^8^^,^^9^ These achievements reflect comparatively well-resourced health systems and established immunization infrastructure.

However, the GCC's unique epidemiological profile is shaped by demographic characteristics that extend well beyond routine childhood coverage statistics. The region hosts one of the world's highest proportions of international migrants relative to the total population: non-nationals constitute approximately 85–88% of residents in Qatar and the UAE and exceed 50% in Bahrain and Kuwait.^10^ Consequently, the numerically dominant share of women of reproductive age are foreign-born individuals from countries with heterogeneous vaccination histories and frequently unavailable immunisation records. Labor migration streams from South Asia, Southeast Asia, and East Africa reinforce this susceptibility complexity.^10^

Simultaneously, the GCC countries are major global travel hubs: Saudi Arabia hosts Hajj and Umrah, the world's largest recurring mass gatherings, drawing pilgrims from more than 180 countries annually,^11^ while international airports in Dubai, Doha, and Abu Dhabi rank among the world's busiest by passenger volume. In combination, these factors sustain conditions for rubella importation and episodic transmission even where domestic burden is low, creating an epidemiological paradox with direct, complex implications for regional elimination strategies.

Despite this relevance, recent rubella and CRS trends across GCC countries have been described primarily through global surveillance systems and broad country-level reporting, rather than through a focused regional synthesis of progress, remaining vulnerabilities, and surveillance implications. The Eastern Mediterranean Regional (EMR) Verification Commission for Measles and Rubella Elimination officially verified the elimination of rubella and measles in Bahrain and Oman in 2019, both having sustained the interruption of endemic transmission. Furthermore, Kuwait and Qatar have confirmed the interruption of transmission but require 36 consecutive months of high-quality surveillance to achieve formal verification.^12^

A comprehensive regional assessment is therefore timely as GCC countries seek to sustain low incidence, prevent the re-establishment of endemic transmission, and strengthen the evidence base for elimination-oriented policy. This study describes recent progress in rubella and CRS control and elimination in GCC countries by examining reported rubella and CRS cases, rubella-containing vaccine coverage, and surveillance structures, and identifies priorities for sustaining progress toward elimination and identifies priorities for sustaining progress toward elimination for the region.

## 2 Methods

### 2.1 Study design and setting

A retrospective ecological study was conducted using aggregated country-level rubella surveillance, congenital rubella syndrome (CRS), and immunisation data from the six GCC member states: Bahrain, Kuwait, Oman, Qatar, Saudi Arabia, and the United Arab Emirates. The study comprised two linked analytical components: (1) a descriptive epidemiological analysis of rubella and CRS patterns, vaccination coverage, and surveillance indicators during 2020–2024; and (2) a scenario-based projection of future reported rubella cases under alternative vaccination coverage assumptions extending from 2025 to 2040.

An ecological design was considered the most appropriate approach because the analysis relied on aggregated national surveillance and immunisation datasets and aimed to evaluate population-level epidemiological patterns and progress toward rubella elimination across GCC countries. This design enabled structured cross-country comparison and policy-relevant interpretation at health-system level.

### 2.2 Data sources

Annual reported rubella cases, rubella incidence per 1,000,000 population, and reported CRS cases were obtained from the WHO Immunization Data Portal and associated WHO downloadable rubella and CRS reporting workbooks.^12^ These data are based on country submissions through the WHO/UNICEF Joint Reporting Form (JRF) or electronic Joint Reporting Form (eJRF) and reflect officially reported figures rather than modelled estimates.^8^

Rubella-containing vaccine first-dose (RCV1) coverage, and second-dose coverage (RCV2) where consistently available, were obtained from the WHO/UNICEF Estimates of National Immunization Coverage (WUENIC), 2024 revision.^8^ WUENIC estimates were used as the primary comparative coverage metric because they apply a standardised methodology that reconciles country-reported administrative data with survey evidence, thereby improving cross-country comparability.

Mid-year population estimates used for incidence calculations were obtained from the United Nations World Population Prospects 2024 online edition.^13^ National rubella vaccination schedules for each GCC country were obtained from WHO vaccination schedule summary resources and cross-referenced with JRF submissions for the study period. Rubella case classifications and CRS case definitions were applied in accordance with WHO vaccine-preventable disease surveillance standards and WHO CRS surveillance guidance.^14^ All data sources were publicly available and accessed in accordance with WHO data use policies.

### 2.3 Case definitions and study variables

Reported rubella cases were defined according to WHO surveillance standards and included laboratory-confirmed, epidemiologically linked, and clinically compatible cases reported through WHO mechanisms; cases discarded following laboratory investigation were excluded.^14^ Imported cases were not excluded unless explicitly distinguished in the source reporting dataset. Reported CRS cases were interpreted according to WHO CRS surveillance case definitions.^14^

The principal variables extracted for the observed analysis were annual reported rubella cases, rubella incidence per 1,000,000 population, annual reported CRS cases, CRS incidence where available, RCV1 coverage, national rubella vaccination schedules, and selected surveillance indicators relevant to elimination-oriented assessment. Four descriptive domains were examined: vaccination coverage, reported rubella incidence, CRS reporting, and surveillance system indicators. This assessment was descriptive in nature and was not intended to constitute formal WHO verification of elimination status.

### 2.4 Data management

All data were extracted into standardised country-by-year matrices. Blank values in WHO downloadable datasets, including empty cells and entries indicating that data were not reported, were recorded as missing or not reported and were not recoded to zero unless a zero value was explicitly present in the source dataset. This distinction was maintained throughout all tables and figures to avoid misrepresenting non-reporting as zero disease burden.

Country names, year labels, and variable formats were harmonised across datasets on rubella cases, incidence, CRS, and vaccination coverage prior to analysis. For pooled GCC estimates, annual country-level counts were aggregated to generate regional totals. Years in which one or more countries had missing rather than reported-zero values were flagged accordingly. Data extraction, cleaning, harmonisation, and descriptive tabulation were conducted primarily using Microsoft Excel for Mac (Microsoft Corp., Redmond, WA, USA).

### 2.5 Descriptive analysis

The observed analysis was descriptive. No inferential statistical modelling or causal estimation was undertaken in this component. Country-year tables were constructed for reported rubella cases and RCV1 coverage (2020–2024), rubella incidence per 1,000,000 population (2020–2024), pooled GCC annual rubella and CRS totals, and a summary assessment of progress toward elimination across the four descriptive domains specified above. Temporal trends were examined graphically using country-level line plots and regional summary figures. A cross-country rubella incidence matrix was used to compare relative burden by country and year. Countries were categorized narratively according to whether they demonstrated persistently zero, sporadic, or sustained non-zero reported rubella incidence during the observed period.

### 2.6 Projection analysis

Future reported rubella cases were projected for 2025–2040 using country-specific Poisson regression models fitted to annual reported rubella case counts for 2020–2024. Calendar year and vaccination coverage were included as explanatory variables, and the natural logarithm of the annual population was included as an offset term to account for changes in population size over time.

This modelling approach was informed by rubella modelling work applied within the Vaccine Impact Modelling Consortium (VIMC) and UK Health Security Agency (UKHSA) rubella modelling frameworks.^15^ However, because the purpose of the present analysis was to describe plausible future reported-case trajectories under alternative programme scenarios, rather than to generate formal age-structured transmission-dynamic estimates of rubella infection or CRS burden, the projections were interpreted as scenario-based estimates of future reported rubella cases.

Four vaccination coverage scenarios were specified a priori. The baseline scenario assumed that coverage would remain broadly consistent with recently observed levels throughout the projection period. The optimistic scenario assumed that coverage would increase to at least 99% in all GCC countries by 2030 and remain sustained thereafter. The pessimistic scenario assumed that coverage would decline by approximately 5–10 percentage points by 2030, reflecting potential programme deterioration. The disruption scenario assumed coverage fluctuations of approximately ±10% across the projection period, reflecting potential programme or external shocks similar to those observed during the COVID-19 pandemic.

Poisson regression was selected as the primary modelling approach because it provided a transparent and parsimonious country-level projection method suitable for short annual surveillance series, including countries with low counts and repeated zero observations. Model-based 95% confidence intervals were derived from the Poisson error structure. Because annual rubella case counts may show outbreak-related variability and potential overdispersion, negative binomial regression was evaluated as a sensitivity analysis. Overdispersion was assessed using the dispersion statistic, and the direction and relative ordering of scenario outputs were compared across model specifications. Projected annual counts were summarised at selected future time points and interpreted as illustrative scenario-based estimates of future reported rubella burden rather than as formal transmission-dynamic forecasts.

### 2.7 Uncertainty and sensitivity analyses

Model-based 95% confidence intervals were interpreted as measures of descriptive uncertainty around projected reported counts. Sensitivity analyses examined the robustness of the projections to alternative model specifications and coverage assumptions. In particular, negative binomial regression was evaluated as an alternative specification where overdispersion relative to the Poisson assumption was suspected. These analyses were used to assess whether the direction and relative ordering of scenario outputs remained stable.

### 2.8 Ethical considerations

This study used aggregated, publicly available secondary data obtained exclusively from WHO immunisation reporting platforms and United Nations (UN) population databases. No individual-level data, patient records, or identifiable information were accessed at any stage. Therefore, formal ethical review and approval were not required, in accordance with applicable institutional and international guidance for secondary analyses of publicly available data. The study was conducted in line with the ethical principles of the Declaration of Helsinki.

## 3 Results

### 3.1 Reported rubella cases and RCV1 coverage, 2020–2024

Marked heterogeneity in reported rubella cases was observed across GCC countries during the study period (Table 1). Bahrain reported zero rubella cases in all five years, with RCV1 coverage remaining stable at 99% throughout the period. Oman also reported zero cases in all years with RCV1 coverage ranging from 97% to 99%; one year of rubella reporting (2022) was missing from the WHO dataset and classified as not reported.

**Table 1:** RCV second-dose (RCV2) coverage (%) in GCC countries by the year of report, and Rubella incidence per 1,000,000 population in GCC countries, by year of report, 2020–2024, and assessment against the WHO elimination threshold.

Country	Metric	2020	2021	2022	2023	2024	Met WHO Elimination Threshold (<1/million in all observed years)
**Bahrain**	Cases (Incidence/M)	0 (0.0)	0 (0.0)	0 (0.0)	0 (0.0)	0 (0.0)	Yes
	RCV1/RCV2 (%)	99/99	99/99	99/99	99/99	99/99	
**Kuwait**	Cases (Incidence/M)	NR	1 (0.2)	3 (0.7)	1 (0.2)	1 (0.2)	Yes
	RCV1/RCV2 (%)	95/93	94/94	99/97	99/98	98/97	
**Oman**	Cases (Incidence/M)	0 (0.0)	0 (0.0)	NR	0 (0.0)	0 (0.0)	Yes
	RCV1/RCV2 (%)	99/99	99/99	97/97	99/99	99/99	
**Qatar**	Cases (Incidence/M)	0 (0.0)	0 (0.0)	0 (0.0)	16 (5.4)	3 (1.0)	No
	RCV1/RCV2 (%)	90/87	99/99	99/99	99/99	99/99	
**Saudi Arabia**	Cases (Incidence/M)	27 (0.9)	86 (2.7)	85 (2.6)	182 (5.5)	96 (2.8)	No
	RCV1/RCV2 (%)	96/95	98/98	98/98	97/97	96/96	
**UAE**	Cases (Incidence/M)	15 (1.6)	3 (0.3)	4 (0.4)	20 (1.9)	25 (2.3)	No
	RCV1/RCV2 (%)	99/97	99/98	98/98	98/97	98/98	

Table showing Rubella surveillance metrics, RCV1 and RCV2 coverage (%), and WHO elimination benchmark assessment across GCC countries, 2020–2024. NR = Not reported in WHO dataset. Incidence per 1,000,000 population. NR = not reported in the WHO source dataset; not recoded as zero. M = Million. RCV1=Rubella containing vaccine 1. GCC = Gulf Cooperated Countries. Sources: WHO Immunisation Data Portal; WUENIC 2024 revision [8, 12].

In contrast, Kuwait reported six cases across the period, with individual annual counts of one case in 2021, three in 2022, one in 2023, and one in 2024; RCV1 coverage increased from 94 to 95% in 2020–2021 to 98–99% in 2022–2024. Qatar reported zero rubella cases in 2020, 2021, and 2022, followed by 16 cases in 2023 and 3 cases in 2024; RCV1 coverage was 90% in 2020, rising to 99% in 2021 and remaining at this level through 2022, 2023, and 2024. The increase in cases in 2023 was not preceded by any documented decline in RCV1 coverage.

Furthermore, Saudi Arabia reported 27 cases in 2020, 86 in 2021, 85 in 2022, 182 in 2023, and 96 in 2024, with RCV1 coverage consistently ranging between 96% and 98% over the five years. The UAE reported 15 cases in 2020, 3 in 2021, 4 in 2022, 20 in 2023, and 25 in 2024, with RCV1 coverage maintained at high levels (98–99%) throughout.

### 3.2 RCV second-dose coverage, 2020–2024

RCV second-dose (RCV2) coverage data were available for all six GCC countries during the study period (Table 2). All six countries operate two-dose MMR schedules, although the timing of dose administration varies slightly by country; in general, both doses are administered between 9 months and 2 years of age 5.

**Table 2:** Pooled GCC reported rubella cases and population-weighted regional incidence, 2020–2024.

Year	Pooled GCC cases	GCC population (millions)	Population-weighted incidence per 1,000,000	Met WHO elimination threshold?
2020	42^a^	57.1	0.9^a^	No
2021	90	57.8	1.6	No
2022	92^a^	58.5	1.6^a^	No
2023	219	59.2	4.7	No
2024	125	59.9	2.6	No

Population denominators: UN World Population Prospects 2024^13^. WHO elimination threshold: <1 per 1,000,000[14]

a Incomplete: Kuwait missing in 2020; Oman missing in 2022.

Bahrain maintained RCV2 coverage at 99% in all five years. Oman likewise sustained high coverage, ranging from 97% to 99% throughout the study period. Kuwait reported RCV2 coverage of 93%-98%, with improvement after 2021. Qatar reported the lowest RCV2 coverage at baseline, with 87% in 2020, followed by an increase to 99% in 2021 that was sustained through 2024. Saudi Arabia reported RCV2 coverage of 95% to 98%, closely mirroring the corresponding RCV1 estimates. The UAE maintained consistently high RCV2 coverage, ranging from 97% to 98%(Table 1).

### 3.3 Rubella incidence per 1,000,000 population, 2020–2024

Country-level rubella incidence per 1,000,000 population is presented in Table 3, alongside an assessment against the WHO rubella elimination threshold of less than one reported case per 1,000,000 population per year 6. Bahrain and Oman recorded an incidence of 0.0 per 1,000,000 in all five years. Kuwait recorded incidence ranging from 0.2 per 1,000,000 in 2021, 2023 and 2024 to 0.7 in 2022. Qatar recorded 0.0 per 1,000,000 in 2020, 2021, and 2022, rising to 5.4 per 1,000,000 in 2023 and declining to 1.0 per 1,000,000 in 2024. Saudi Arabia recorded incidence of 0.9 per 1,000,000 in 2020, 2.7 in 2021, 2.6 in 2022, 5.5 in 2023, and 2.8 in 2024. The UAE recorded incidence of 1.6 per 1,000,000 in 2020, 0.3 in 2021, 0.4 in 2022, 1.9 in 2023, and 2.3 in 2024.

**Table 3:** Summary of rubella and CRS control indicators by GCC member state, 2020–2024.

Country	Total rubella cases	Peak incidence per 1,000,000	Met WHO threshold (all years)?	Total CRS cases	Mean RCV1 2020–2024 (%)	Mean RCV2 2020–2024 (%)	Overall progress status
**Bahrain**	0	0.0	Yes	0	99.0	99.0	Elimination-consistent
**Kuwait**	6	0.7	Yes	0	97.0	94.6	Strong progress; sporadic transmission
**Oman**	0^a^	0.0	Yes	0	98.6	98.6	Elimination-consistent
**Qatar**	19	5.4	No	0	97.2	96.6	Moderate progress; outbreak-prone
**Saudi Arabia**	476	5.5	No	0	97.0	96.8	Partial progress; persistent burden
**UAE**	67	2.3	No	0	98.4	97.6	Moderate progress

a One missing rubella reporting year (2022). CRS zero values reflect officially reported figures only.

Bahrain and Oman met the WHO elimination incidence threshold in all observed years. Kuwait met the threshold in all reported years. Qatar met the threshold in 2020–2022 but exceeded it in 2023 and recorded incidence at the threshold boundary in 2024. Saudi Arabia exceeded the threshold in every year of the study period. The UAE exceeded the threshold in 2020, 2023, and 2024, while remaining below the threshold in 2021 and 2022 (Table 1).

### 3.4 Pooled GCC rubella cases and regional incidence, 2020–2024

Pooled GCC annual reported rubella cases and population-weighted regional incidence estimates are presented in Table 2. Annual reported cases ranged from 42 in 2020 to 219 in 2023, before declining to 125 in 2024. Population-weighted regional incidence ranged from 0.9 per 1,000,000 in 2020 to a peak of 4.7 per 1,000,000 in 2023, then declined to 2.6 per 1,000,000 in 2024. The pooled GCC regional incidence remained above the WHO programmatic benchmark of less than 1 reported case per 1,000,000 population in every study year.

Pertinently, pooled estimates for 2020 and 2022 should be interpreted cautiously because country-year data were incomplete in those years, with missing rubella reports for Kuwait in 2020 and Oman in 2022. Even with this caveat, the overall pattern indicates that the GCC did not sustain a consistently low regional rubella burden during the observed period, with the highest regional burden occurring in 2023.

### 3.5 CRS reporting, 2020–2024

No CRS cases were reported in any GCC member state during 2020–2024 (Table 3). This pattern was consistent across all available country-year observations, except for one missing CRS observation for Kuwait in 2020. Although the absence of reported CRS cases is reassuring, it should be interpreted cautiously, as passive surveillance may under-ascertain the true CRS burden, particularly when case recognition, laboratory confirmation, and reporting pathways are incomplete.

Table 3 summarises the main rubella and CRS control indicators across the GCC countries. Bahrain and Oman showed the strongest elimination-oriented profiles, with zero reported rubella and CRS cases, no peak incidence, and very high mean RCV1 and RCV2 coverage. Kuwait also maintained high coverage and remained below the WHO incidence benchmark in all reported years, although sporadic non-zero rubella transmission was observed. Qatar, Saudi Arabia, and the UAE maintained high vaccine coverage but reported non-zero rubella burden, indicating uneven progress toward elimination-oriented control across the region.

### 3.6 Scenario-based projections of annual reported rubella cases, 2026–2055

Scenario-based projections of annual reported rubella cases for selected future time points are presented in Table 4, and the corresponding scenario interpretation is summarised in Table 5. Fig. 1 illustrates the optimistic and pessimistic trajectories across the projection period.

**Table 4:** Projected annual reported rubella cases in GCC member states under four vaccination coverage scenarios, selected years 2030–2040.

Year	Country	Baseline, n (95% CI)	Optimistic, n (95% CI)	Pessimistic, n (95% CI)	Disruption, n (95% CI)
2030	Bahrain	0 (0.0–3.7)	0 (0.0–3.7)	1 (0.0–5.6)	1 (0.0–5.6)
2030	Kuwait	2 (0.2–7.2)	1 (0.0–5.6)	4 (1.1–10.2)	3 (0.6–8.8)
2030	Oman	0 (0.0–3.7)	0 (0.0–3.7)	1 (0.0–5.6)	1 (0.0–5.6)
2030	Qatar	6 (2.2–13.1)	2 (0.2–7.2)	12 (6.2–21.0)	8 (3.5–15.8)
2030	Saudi Arabia	100 (81.4–121.6)	50 (37.1–65.9)	150 (127.0–176.0)	120 (99.3–143.6)
2030	UAE	20 (12.2–30.9)	5 (1.6–11.7)	60 (45.8–77.1)	30 (20.2–42.8)
2035	Bahrain	0 (0.0–3.7)	0 (0.0–3.7)	1 (0.0–5.6)	1 (0.0–5.6)
2035	Kuwait	2 (0.2–7.2)	0 (0.0–3.7)	5 (1.6–11.7)	3 (0.6–8.8)
2035	Oman	0 (0.0–3.7)	0 (0.0–3.7)	1 (0.0–5.6)	1 (0.0–5.6)
2035	Qatar	8 (3.5–15.8)	1 (0.0–5.6)	15 (8.4–24.8)	10 (4.8–18.4)
2035	Saudi Arabia	110 (90.4–132.8)	20 (12.2–30.9)	180 (154.6–208.4)	130 (108.5–154.9)
2035	UAE	15 (8.4–24.8)	0 (0.0–3.7)	70 (54.5–88.5)	25 (16.2–36.9)
2040	Bahrain	0 (0.0–3.7)	0 (0.0–3.7)	1 (0.0–5.6)	1 (0.0–5.6)
2040	Kuwait	2 (0.2–7.2)	0 (0.0–3.7)	6 (2.2–13.1)	3 (0.6–8.8)
2040	Oman	0 (0.0–3.7)	0 (0.0–3.7)	1 (0.0–5.6)	1 (0.0–5.6)
2040	Qatar	10 (4.8–18.4)	0 (0.0–3.7)	20 (12.2–30.9)	12 (6.2–21.0)
2040	Saudi Arabia	120 (99.3–143.6)	0 (0.0–3.7)	200 (173.2–229.7)	140 (117.6–165.5)
2040	UAE	10 (4.8–18.4)	0 (0.0–3.7)	80 (63.5–99.3)	20 (12.2–30.9)

95% CI = 95% confidence interval derived from Poisson error structure. For countries with zero or near-zero observed counts (Bahrain, Oman), wide confidence intervals reflect extrapolation from a series with minimal informative variance and should be interpreted with caution.

**Table 5:** Vaccination coverage scenario definitions and projected regional outcomes, 2026–2055.

Scenario	Coverage assumption	Projected regional outcome
**Baseline**	Coverage maintained at recent observed levels throughout	Bahrain and Oman remain at zero reported cases; Kuwait maintains a very low burden; Qatar and Saudi Arabia continue to contribute persistent non-zero burden; UAE shows a declining but non-zero trajectory
**Optimistic**	All countries reach ≥99% RCV1 and RCV2 by 2030, sustained thereafter	All GCC countries reach zero projected cases by 2040
**Pessimistic**	Coverage declines 5–10 percentage points by 2030	Progressive increase in reported rubella cases across affected countries, with the largest increases projected in Saudi Arabia, followed by the UAE and Qatar
**Disruption**	Coverage fluctuates ±10% due to programmatic or external shocks	Intermediate fluctuating burden across affected countries, generally between baseline and pessimistic projections

**Fig. 1:**
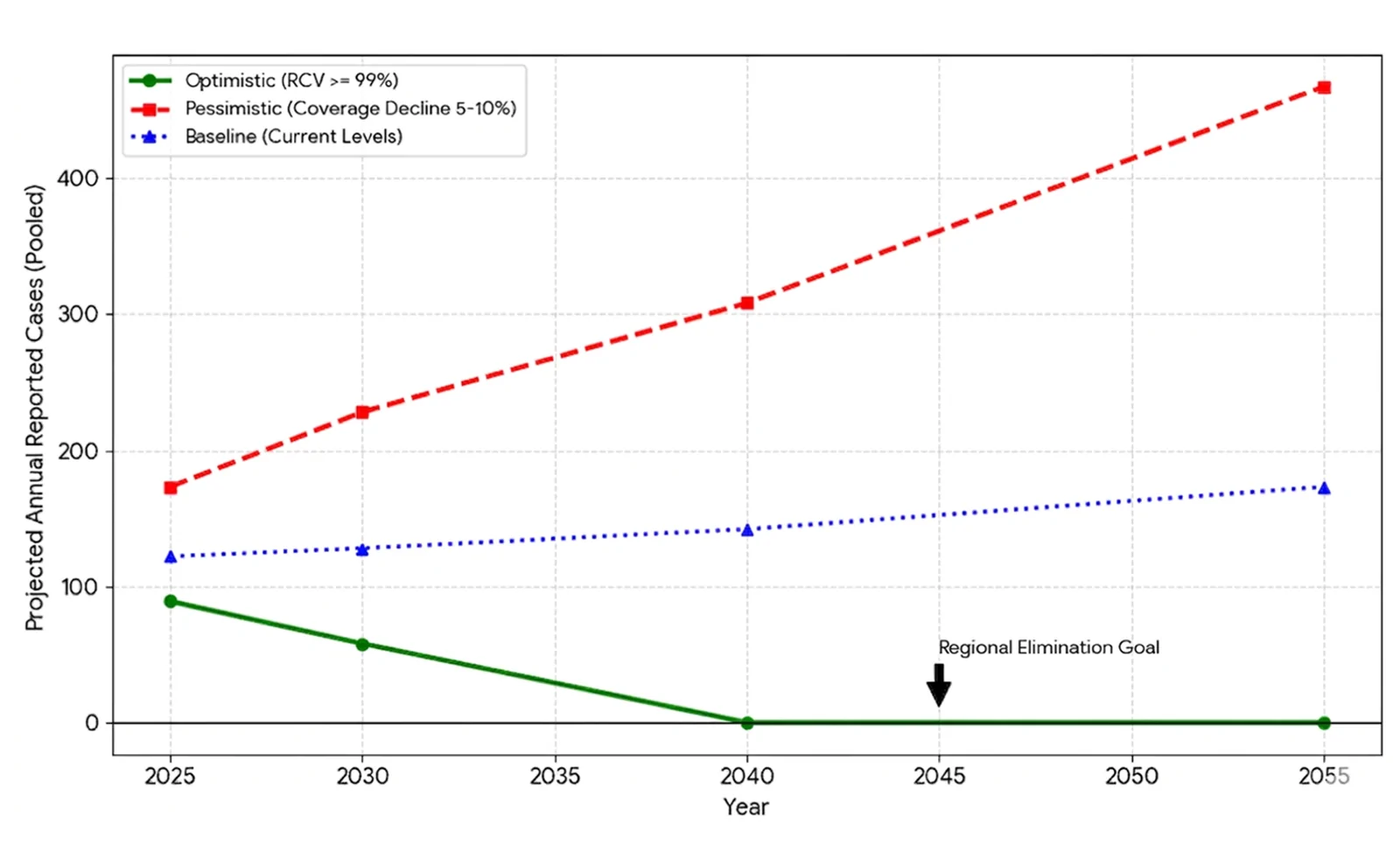
The optimistic and the pessimistic approaches in GCC countries (2025-2055).

Under the baseline scenario, Bahrain and Oman were projected to remain at zero reported cases through 2040, while Kuwait was projected to sustain a low burden of approximately two cases annually. Qatar was projected to increase gradually from 6 cases in 2030 to 10 in 2040, and Saudi Arabia remained the principal contributor to the projected regional burden, rising from 100 cases in 2030 to 120 in 2040. In contrast, the UAE showed a declining baseline trajectory, from 20 projected cases in 2030 to 10 in 2040.

Under the optimistic scenario, in which vaccination coverage improved to at least 99% by 2030 and was sustained thereafter, Bahrain and Oman maintained a zero-case profile throughout. Kuwait was projected to reach zero cases from 2035 onward, while Qatar, Saudi Arabia, and the UAE were all projected to reach zero reported cases by 2040.

Under the pessimistic scenario, defined by a 5–10 percentage point decline in coverage by 2030, projected burden increased across all currently affected countries. By 2040, projected annual counts were 200 in Saudi Arabia, 80 in the UAE, 20 in Qatar, and 6 in Kuwait. Under this scenario, Bahrain and Oman were each projected to report 1 case annually.

Under the disruption scenario, incorporating programme or external shocks with approximately ±10% fluctuations in coverage, projected case counts were intermediate between the baseline and pessimistic scenarios across most countries and years. These estimates should be interpreted as scenario-based trajectories of future reported cases under alternative coverage assumptions rather than as formal age-structured transmission-model estimates of rubella infection or CRS burden.

## 4 Discussions

This regional synthesis of rubella and CRS control across the six GCC countries between 2020 and 2024 revealed a polarised epidemiological landscape characterised by three principal patterns: sustained, elimination-consistent zero incidence in Bahrain and Oman; persistent low-level transmission in Saudi Arabia and the UAE despite consistently high two-dose RCV coverage exceeding 95%; and episodic, outbreak-prone transmission in Qatar, which occurred without any documented programmatic decline in vaccination coverage. Collectively, these findings demonstrate that high childhood immunisation coverage did not uniformly translate into interruption of transmission across all GCC member states.

Rubella elimination requires more than low annual incidence alone. Under WHO frameworks, elimination is defined as the absence of endemic rubella virus transmission in a defined geographical area in the presence of a well-performing surveillance system, and formal verification requires epidemiological, laboratory, and surveillance evidence beyond routine coverage estimates or low annual case counts alone. 1, 2, 3 Against this standard, the present analysis suggests substantial but uneven progress across the GCC during 2020–2024 and is therefore more consistent with progress toward elimination-oriented control than with formal elimination verification.

While the pooled GCC countries incidence did not meet the WHO elimination threshold of <1 per 1,000,000 in any study year, peaking at 4.7 per 1,000,000 in 2023. This positions the GCC countries in an intermediate epidemiological stratum: beyond the phase of endemic transmission driven by under-immunisation, but not yet at the sustained low-incidence level associated with verified regional elimination.

Three principal patterns emerged. First, Bahrain and Oman showed the strongest elimination-oriented profiles, with zero reported rubella incidence in all reported years and consistently high routine vaccine coverage. Second, Saudi Arabia contributed the largest share of the regional burden and reported non-zero rubella cases in every study year despite sustained high routine coverage. Third, Qatar experienced a marked increase in 2023 despite maintained vaccine uptake, indicating that strong childhood programme performance alone does not eliminate the possibility of episodic transmission. Across the region, no CRS cases were reported, although this must be interpreted cautiously because passive CRS surveillance is known to underestimate true burden where clinical recognition, laboratory confirmation, and reporting pathways are incomplete.^1^^,^^2^^,^^14^^,^^16^ Progress toward rubella elimination provides substantial epidemiological momentum for regional measles elimination. Because rubella has a lower basic reproduction number ($R_{0}∼6‐8$) than measles ($R_{0}∼12‐18$), interrupting endemic rubella transmission offers an achievable proof of concept for the sensitivity of integrated fever-rash surveillance, cold chain resilience, and laboratory confirmation capacity across the GCC states.

These findings should be interpreted within the broader context of substantial but incomplete global progress. Reported rubella cases declined from 93,816 in 2012 to 17,407 in 2022, while the number of countries introducing rubella-containing vaccines increased from 132 to 175 over the same period 4. Rubella and CRS elimination remain embedded within the WHO Immunisation Agenda 2030 5. However, progress remains uneven across and within WHO regions, and low-incidence settings remain vulnerable to recurrent transmission when susceptibility pockets persist. In this respect, the GCC states appears to occupy an intermediate epidemiological position: clearly beyond the high-burden phase associated with weak immunisation systems, but not yet uniformly at the sustained low-incidence level associated with verified elimination.^2^^,^^3^

The central interpretive lesson from the GCC countries data is that high national vaccine coverage did not uniformly translate into zero reported burden. This was particularly evident in Saudi Arabia and, episodically, in Qatar and the UAE. Such patterns suggest that routine childhood coverage, although essential, may not fully reflect population-level susceptibility. In addition to adult susceptibility and importation risk, persistent low-level transmission in high-coverage settings may also reflect subnational heterogeneity in vaccine uptake, clustering of unvaccinated individuals, and uneven surveillance sensitivity within and across countries. National routine coverage estimates should therefore not be interpreted as equivalent to population immunity across all age and demographic groups.^1^^,^^6^ Furthermore, global rubella policy updates recommending universal RCV introduction under the Immunization Agenda 2030 are anticipated to introduce rubella vaccination into remaining non-vaccinating source countries (e.g., in sub-Saharan Africa) by 2030. With the increase in population immunity globally, regional viral circulation will drop with less external importation pressure into GCC states. This will lead to reduced incidence rates. While all GCC states provide childhood EPI vaccines free of charge to non-citizen children in public sector institutions, many expatriate dependents utilize private health facilities where immunisation tracking and reporting to central registries remain variable. Additionally, adult migrant workers from diverse geographical origins often lack vaccination records, leaving possible pockets of adult susceptibility.

This issue is especially relevant in the GCC countries because the region is characterised by intense international mobility and a large foreign-born workforce. In such settings, national childhood coverage may coexist with susceptible adult subpopulations whose prior vaccination histories are heterogeneous. The implication is not that migration alone explains residual transmission, but rather that population mobility may complicate rubella elimination when susceptible adults remain outside the reach of routine childhood immunisation indicators.^7^^,^^8^ At the same time, the country-reported datasets used in this analysis did not consistently distinguish imported, import-related, and locally acquired rubella cases across all country-years, limiting direct inference about transmission origin.^1^^,^^17^^,^^18^ The role of migrant and mobile populations should therefore be interpreted as epidemiologically plausible in the GCC countries context rather than directly demonstrated by the present data.

A useful convergent comparison comes from an antenatal susceptibility study conducted among pregnant women in North London between 1996 and 1999, which found that rubella susceptibility remained low overall but was higher among women born outside the United Kingdom than among UK-born women.^19^ This finding is relevant because it shows that strong routine childhood immunisation programmes do not necessarily eliminate immunity gaps in women of reproductive age. A related clinical surveillance report from the United Kingdom showed that CRS can still occur in high-coverage settings among infants born to non-immune immigrant mothers.^19^ These studies converge with the present GCC countries findings by showing that high childhood coverage may coexist with adult susceptibility and residual congenital risk. They differ, however, in that the UK evidence was based on direct maternal susceptibility and case-based clinical observation, whereas the present GCC countries study relied on aggregated surveillance data and could not directly measure immunity profiles in adult women.^18^^,^^19^

A second convergent comparison comes from a national epidemiological surveillance analysis from Singapore covering 1991–2007, which documented ongoing rubella epidemiology in a setting with strong routine programme performance.^20^ The transferable lesson is that low reported burden in a well-performing immunisation programme does not necessarily imply absence of susceptibility in all adult population groups. This is similar to the GCC pattern, particularly in Qatar, Saudi Arabia, and the UAE, where high routine coverage coexisted with continued reported transmission. The difference is that the Singapore analysis was a long-term national surveillance review, whereas the present GCC countries study focused on a shorter regional period and explicitly examined elimination-oriented indicators.^21^

A more divergent comparison comes from the Americas. A regional implementation analysis of rubella and CRS elimination in the Americas described how elimination was achieved through the combination of high routine coverage, supplementary immunisation activities, and surveillance systems capable of documenting interruption of endemic transmission.^16^ The key contrast with the GCC countries is that the Americas complemented childhood vaccination with broader population strategies, including campaigns that reached adolescents and adults, while also building verification-ready surveillance. The relevance for the GCC countries are therefore, not that the same pathway can be copied directly, but that high childhood coverage alone may be insufficient to achieve verifiable elimination where susceptible adults remain outside the routine infant schedule.^16^

A further convergent comparison comes from Japan. A retrospective sero-epidemiological analysis and related epidemic investigation from the 2012–2014 rubella epidemic showed that substantial outbreaks can occur despite high childhood vaccine coverage when susceptible adult cohorts remain.^22^^,^^23^ In Japan, susceptibility was concentrated mainly in adult men who had not been covered by earlier vaccination policies. This differs from the GCC countries, where the likely sources of residual susceptibility are more heterogeneous and may include mobile or foreign-born populations. Nonetheless, the underlying mechanism is similar: strong childhood coverage does not fully protect a population if susceptible adult cohorts remain large enough to sustain transmission.^22^^,^^23^

The absence of reported CRS cases across the GCC countries should also be interpreted with caution. Passive CRS surveillance is well recognised to underestimate true burden, especially where clinical recognition, laboratory confirmation, and reporting pathways are incomplete.^1^^,^^14^ A hospital-based active surveillance study with retrospective record review in Kumasi, Ghana identified CRS cases during an otherwise poorly documented epidemic and showed that clinically important congenital infection may remain unrecorded in routine passive systems.^24^ A global burden modelling analysis also demonstrated that reported CRS is likely to represent only a fraction of true CRS occurrence in many settings.^25^ These studies converge with the caution needed in interpreting zero reported CRS in the GCC countries. They differ from the present study in that they used active or model-based approaches specifically aimed at revealing hidden congenital burden, whereas the GCC countries analysis relied on reported surveillance outputs alone.^24^^,^^25^

Published data from Oman provide additional context. A community-based hearing impairment study and a neonatal hearing screening programme report documented organised approaches to hearing assessment in Oman.^26^^,^^27^ These do not prove absence of CRS, but they suggest somewhat greater potential for detecting congenital sensorineural hearing deficits than would be expected in a setting relying solely on passive clinical reporting. This makes the Omani zero-case profile more plausible than it might otherwise appear, although comparable evidence on linked CRS ascertainment systems was not available for the other GCC countries in the present dataset.^8^

Taken together, these comparisons suggest that the GCC findings are broadly convergent with evidence from other high-coverage settings showing that residual adult susceptibility may persist despite strong childhood immunisation programmes, but divergent from regions such as the Americas where elimination was achieved through broader adult-targeted strategies and stronger verification-ready surveillance.^8^^,^^14^ The policy implication is therefore clear, maintaining high routine childhood coverage remains essential, but it is unlikely to be sufficient on its own.

### 4.1 Policy implications

Several practical implications follow from these findings. First, Bahrain and Oman appear closest to an elimination-compatible profile and should focus on sustaining high routine coverage, maintaining surveillance sensitivity, and strengthening documentation needed for future verification pathways. 1, 2, 3 Second, countries with persistent or episodic non-zero burden, particularly Saudi Arabia, Qatar, and the UAE, may require more systematic approaches to identifying and vaccinating susceptible adults, especially women of reproductive age. Existing service points such as postpartum care, preconception services, and premarital care may provide practical opportunities for this.^2^ mmunization policies must be expanded beyond routine infant schedules to incorporate targeted occupational and pre-employment health protocols. These include voluntary adult RCV catch-up during entry medical examinations for expatriate labor forces, pre-travel vaccination recommendations for outbound travelers, and clear vaccination verification protocols for mass gathering attendees (such as Hajj and Umrah pilgrims).

Third, CRS surveillance should be strengthened across the region. Greater laboratory support, clearer clinical referral pathways, and improved linkage with congenital anomaly detection systems would make zero-case findings more interpretable and strengthen the evidence base for elimination-oriented control.^1^^,^^14^ Fourth, case classification and molecular epidemiological capacity need to improve. Distinguishing endemic transmission from repeated importation is central to elimination assessment, and more consistent documentation of importation status would greatly strengthen future regional analyses.^1^^,^^4^^,^^14^^,^^16^ Finally, the projection findings suggest that sustained high coverage remains the main determinant of future reported burden. Under optimistic coverage assumptions, several countries move toward zero or near-zero reported cases, whereas reduced or unstable coverage is associated with persistence or resurgence of reported transmission.^22^

### 4.2 Study limitations

Several limitations should be considered when interpreting these findings. First, all data were derived from official WHO reporting platforms reflecting country submissions and are therefore subject to differences in surveillance sensitivity, testing practices, reporting completeness, and case classification across countries^.17,18^ Because the analysis relied on country-reported surveillance data, observed differences between countries may reflect variation in case detection and reporting as well as true epidemiological differences. Second, blank values in source files were treated as missing rather than zero, which may have produced conservative pooled regional totals in years with incomplete reporting. Third, the available datasets did not consistently distinguish imported, import-related, and locally acquired rubella cases, limiting inference about transmission origin. Fourth, the analysis did not incorporate sero-prevalence data, molecular epidemiology, age-disaggregated case data, subnational vaccine coverage, or migrant-specific immunity data, all of which would be required for a fuller understanding of residual susceptibility. Fifth, reported CRS counts were derived from surveillance systems that may under-ascertain true congenital infection burden, especially where passive detection predominates. Finally, the scenario-based projections were designed as policy-illustrative trajectories of reported cases under alternative coverage assumptions and did not explicitly model herd immunity thresholds, age structure, population turnover, or importation dynamics. They should therefore be interpreted as illustrative reported-case scenarios rather than as formal transmission-dynamic forecasts.

Finally, the scenario-based Poisson projections represent policy-illustrative trajectories of reported cases predicated on childhood vaccination coverage, rather than age-structured dynamic transmission models. Consequently, they do not account for external importation velocity, age-stratified adult mixing patterns, or population turnover.

## 5 Conclusion

The GCC has achieved substantial progress in rubella control, with the strongest elimination-oriented profiles observed in Bahrain and Oman. However, the region as a whole did not consistently display the low-incidence pattern expected of a clearly verified elimination setting, and Saudi Arabia, Qatar, and the UAE continued to show evidence of residual vulnerability. These findings should be interpreted as evidence of progress toward elimination-oriented control rather than formal elimination verification. Sustained regional progress will require not only high childhood vaccine coverage, but also stronger surveillance, improved case classification, more sensitive CRS detection, and better identification of susceptible adult populations. Regional elimination appears achievable, but it will depend on coupling strong routine immunisation with surveillance systems capable of distinguishing importation from continued local transmission and of supporting future verification efforts.

## CRediT authorship contribution statement

**Salah T. Al Awaidy:** Writing – review & editing, Writing – original draft, Visualization, Validation, Supervision, Software, Methodology, Investigation, Formal analysis, Data curation, Conceptualization. **Amani Abdelmoniem Mahmoud Mustafa:** Writing – review & editing, Writing – original draft, Methodology, Investigation, Data curation, Conceptualization. **Sumaiya Al Aamri:** Writing – review & editing, Writing – original draft, Supervision, Methodology, Investigation, Conceptualization. **Jameela alsalman:** Writing – review & editing, Writing – original draft, Supervision, Methodology, Investigation, Conceptualization. **Hamad Eid H.A. Al-Romaihi:** Writing – review & editing, Writing – original draft, Supervision, Methodology, Investigation, Data curation, Conceptualization. **Khalid Hamid Elawad:** Writing – review & editing, Writing – original draft, Validation, Investigation, Conceptualization. **Mahim Albalushi:** Writing – review & editing, Supervision, Methodology, Investigation, Data curation, Conceptualization. **Rana Al Maghrabi:** Writing – review & editing, Writing – original draft, Methodology, Investigation, Conceptualization. **Ahmed AlHammadi:** Writing – review & editing, Writing – original draft, Methodology, Investigation, Conceptualization. **Parvaiz A. Koul:** Writing – review & editing, Writing – original draft, Validation, Software, Methodology, Investigation, Data curation, Conceptualization. **Ozayr Mahomed:** Writing – review & editing, Writing – original draft, Validation, Supervision, Methodology, Investigation, Formal analysis, Data curation, Conceptualization.

## Funding

No funding for this research.

## Declaration of competing interest

The authors declare that they have no known competing financial interests or personal relationships that could have appeared to influence the work reported in this paper.

## Data Availability

Data will be made available on request.

## Abbreviations

CRS: Congenital Rubella Syndrome
GCC: Countries Gulf Cooperation Council countries
RCV: rubella containing-vaccines
MMR: measles-mumps-rubella
WUENIC: WHO/UNICEF Estimates of National Immunization Coverage
JRF: Joint Reporting Form
VIMC: Vaccine Impact Modelling Consortium
UKHSA: UK Health Security Agency
UAE: United Arab Emirates
